# Abdominal aortic aneurysm with horseshoe kidney with central renal artery: A vascular dilemma

**DOI:** 10.1016/j.jvscit.2024.101579

**Published:** 2024-07-22

**Authors:** Emma Morel, S Christopher Frontario, Nakul Rao, Thomas Bernik

**Affiliations:** Vascular Surgery Department, Englewood Heath, Englewood, NJ

**Keywords:** Abdominal aortic aneurysm, EVAR, Horseshoe kidney

## Abstract

Abdominal aortic aneurysm with concomitant horseshoe kidney is exceedingly rare. Although open repair was previously the treatment, endovascular aortic repair has become an increasingly popular option. In the current endovascular era, complex aortic pathologies are treatable with selective use of multiple advanced techniques. We present a unique case involving complex endovascular repair of abdominal aortic aneurysm complicated by presence of horseshoe kidney with central renal artery in a patient where an open approach was prohibited.

Horseshoe kidney (HSK) is a rare congenital fusion defect and is uncommonly associated with abdominal aortic aneurysm (AAA).^1^ Surgical management depends on location of AAA, HSK, and aberrant vasculature. Historically, open repair was the standard treatment, but endovascular aortic repair (EVAR) has become a more popular option due to the morbidity associated with open surgery.^2^ We present a unique case of AAA with HSK that was successfully treated with complex endovascular repair. Proper consent was obtained from the patient to publish this case report and imaging.

## Case presentation

**Fig 1:**
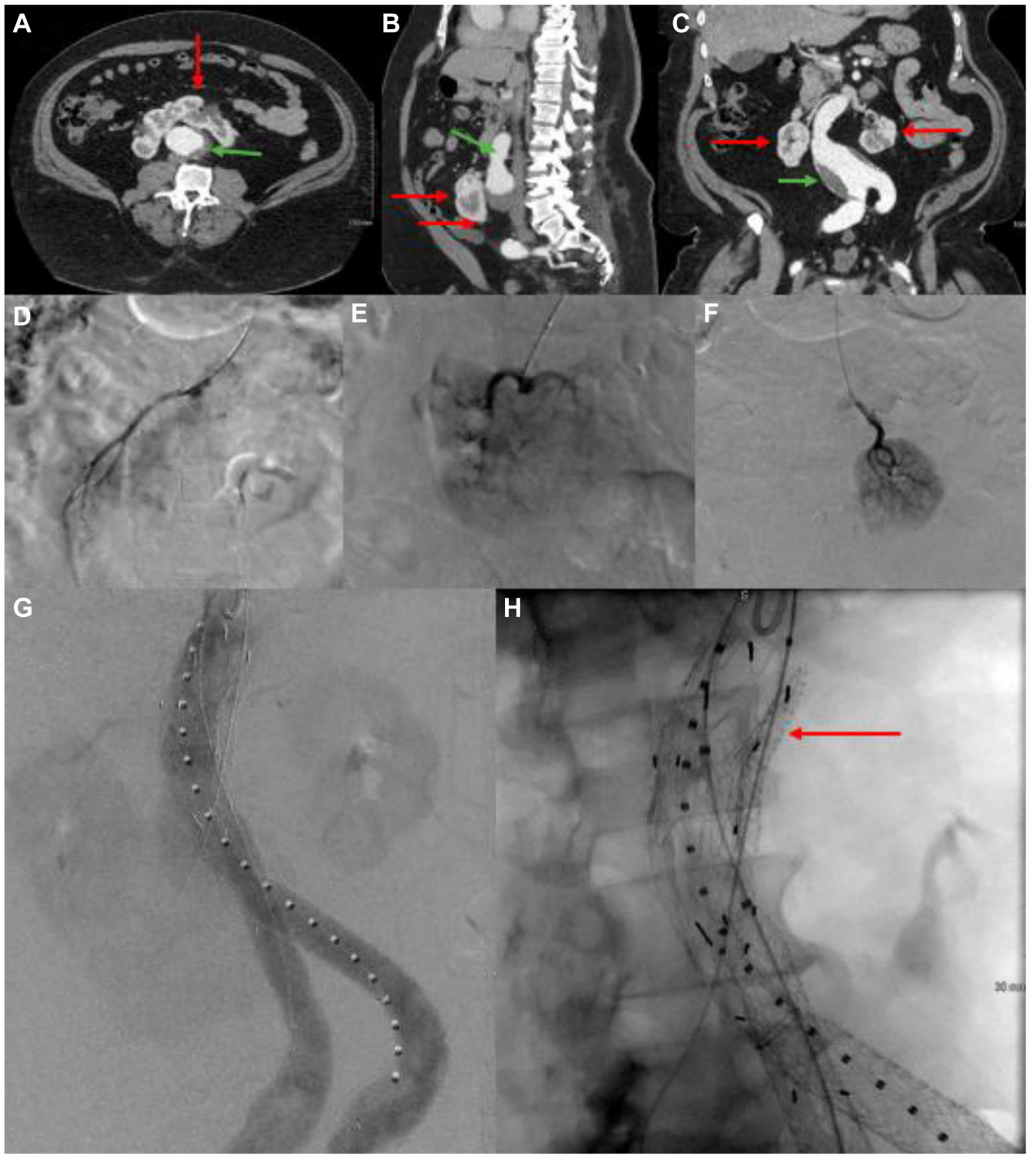
**A-C**, Computed tomography (*CT*) imaging of abdominal aortic aneurysm (*AAA*) (*green arrow*) and horseshoe kidney (*HSK*) (*red arrow*); **D,** Right renal artery; **E,** Anterior renal trunk artery; **F,** Left renal artery; **G,** Bifurcated endograft, aortic cuff, renal snorkel (*red arrow*), iliac extension, and flared limbs; **H,** Type II endoleak vs gutter leak.

A 62-year-old morbidly obese (body mass index, 53 kg/m^2^) African American male was referred for elective evaluation of AAA. His medical history included severe obstructive sleep apnea, cerebral vascular accident with residual aphasia, hypertension, and no prior surgical history. Computed tomography (CT) angiography demonstrated a 5.6-cm infrarenal AAA with concomitant bilateral iliac artery aneurysms and a central horseshoe kidney with aberrant vasculature with infrarenal neck length of 19 mm (Fig 1, *A–C*). His preoperative workup included split renal function and a diagnostic aortogram, which demonstrated 20% right and left renal artery peripheral perfusion (Fig 1, *D* and *E*, respectively) and a dominant short anterior renal artery trunk supplying the central 50% of the renal parenchyma (Fig 1, *F*).

Multiple operative strategies were explored along with all potential pitfalls. These included open repair via transabdominal vs retroperitoneal approach with or without isthmus division. Endovascular possibilities included fenestrated endograft, anterior renal trunk snorkel, iliac extension with flared limbs, coiling the hypogastric with extension into the external iliac, and use of iliac branch endoprostheses. There were no commercially available fenestrated grafts compatible with the ultimate build out of the repair, given the infrarenal aortic length and iliac aneurysms. Additionally, a hybrid option was constructed with open retrograde renal bypass followed by endograft placement. Ultimately, an endovascular option was implemented with anterior renal artery snorkel through brachial cutdown, bifurcated aortic graft placement, iliac extension limbs, bilateral flared iliac limbs, and aortic extension cuff (Fig 1, *G* and *H*). The renal snorkel was particularly challenging, given the short central renal artery, but with a 1-cm landing zone, 10% oversizing, and apposition by the aortic graft, the renal component was felt to be durable. Angiography confirmed what appeared to be a delayed type II endoleak vs gutter leak, which fully resolved a week after discharge. The patient has been followed at 1 week, 1 month, 6 months, and 12 months with findings of a patent graft without endoleak on duplex and CT and intact renal function.

## Discussion

HSK is a rare renal anomaly found in 0.25% of the population.^1^ Although AAA is a common vascular pathology, only 0.12% of patients who undergo repair have concomitant HSK.^2^ Considering the complexity of the dual pathology, special care must be taken regarding preoperative planning, operative approach, and postoperative surveillance to ensure good outcomes.

Preoperative planning should involve several imaging modalities including CT, duplex, angiography, and split renal function. Duplex ultrasound is often the first diagnostic study for AAA. In the setting of aberrant anatomy, duplex alone is insufficient for preoperative planning.^3^ CT scan provides the most accurate evaluation of aortic aneurysms and identification of other intra-abdominal abnormalities.^4^ Lastly, conventional angiography and nuclear medicine split renal function assessment provides an excellent road map for graft placement with maximal preservation of renal function. Given that most HSK have variant arterial anatomy, a thorough assessment is vital to preserving renal function and preventing parenchymal loss.^4^ These arterial variants may not have significant collateral flow, which risks functional tissue loss if covered by the endograft or divided during open surgical repair.^5^

Specific challenges should be considered in preoperative planning for a patient with AAA and HSK: location of the horseshoe kidney with its isthmus, location of ureters, and variable vasculature.^6^ Division of the isthmus is often necessary for adequate aortic exposure with an open approach.^1^ Management of the isthmus remains highly controversial due to possible need for symphysiotomy, aberrant ureter location, and risk for tissue ischemia. The resulting complications include urinary leaks, urinoma formation, infection, hemorrhage, fistula formation, and parenchymal ischemia leading to postoperative renal insufficiency.^7^ Considering the complications, many authors agree symphysiotomy should be avoided unless the isthmus precludes aortic reconstruction.^8^

**Fig 2:**
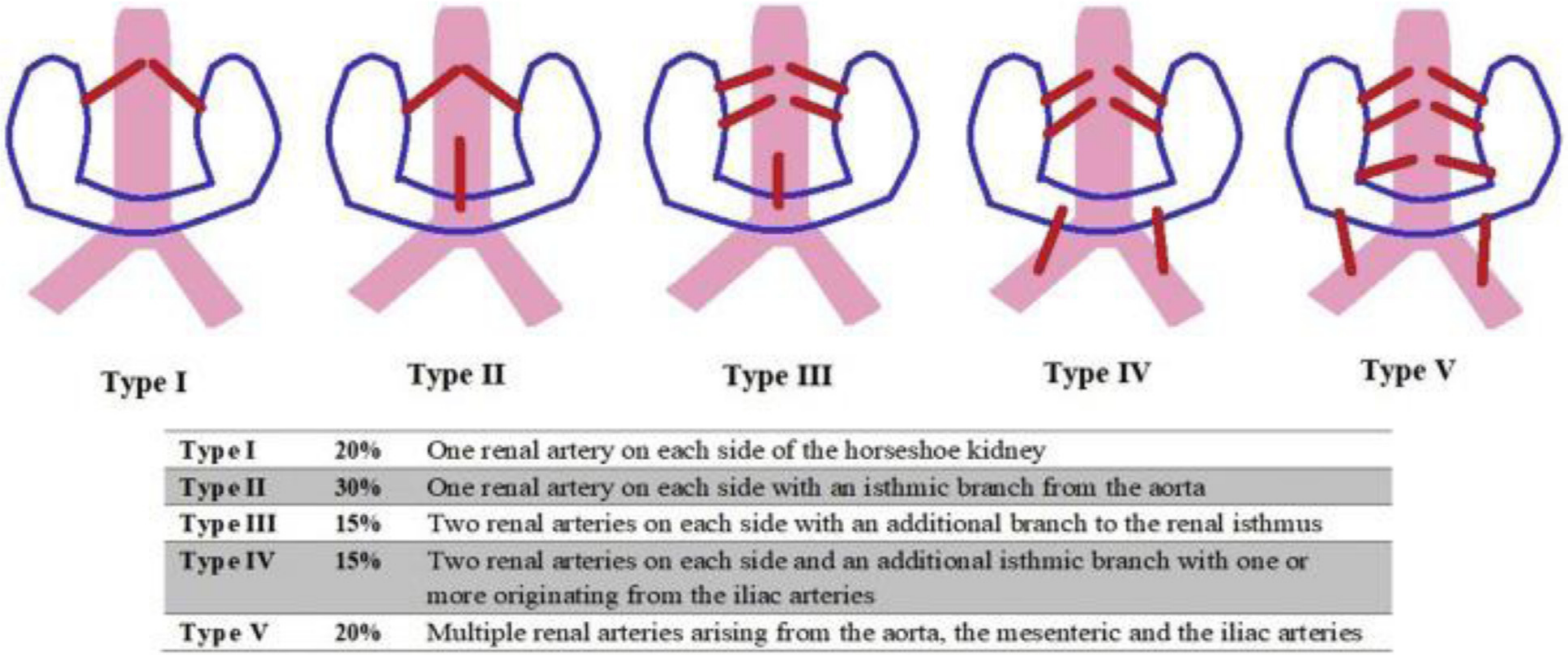
Eisendrath classification system of horseshoe kidney (*HSK*) vasculature.

Eisendrath et al classified the aberrant HSK vasculature (Fig 2), which each pose unique challenges in maintaining solid organ perfusion for both open and endovascular repair.^9^ Ruppert and colleagues described a systematic approach of anomalous vasculature when planning for EVAR with graft coverage of non-dominant accessory renal arteries less than 3 mm in diameter, given that the isthmus can be covered, whereas coverage of accessory renal arteries greater than 3 mm in diameter create risk for type II endoleak.^10^ Sharma et al described successfully employing chimney strategy to ameliorate challenges posed by dominant accessory renal arteries.^6^ Customized fenestrated endografts are another viable option to preserve renal function but are limited due to manufacturer and anatomic constraints.^8^ Collectively, these challenges make aneurysm repair in the setting of HSK a complex vascular dilemma.

Open and endovascular approaches to AAA with associated HSK have been described in the literature. Historically open repair was favored. However, endovascular repair has gained acceptance as more advanced techniques develop.^11^ Open approaches include transperitoneal and left retroperitoneal exposures, with a transperitoneal approach offering optimal aneurysmal exposure and expeditious vascular control, whereas a left retroperitoneal approach excludes the isthmus and urinary system but limits full distal control.^7^ EVAR is an excellent option for patients who cannot tolerate an open repair and avoid interference with the isthmus and collecting system. EVAR limitations include proximal and distal seal zones, neck angulation, and anomalous vascular anatomy, which may lead to parenchymal loss with graft coverage. Advances in endovascular technology and techniques including custom fenestrated grafts, snorkels, and chimneys have been described successfully.^6^

These patients require intense postoperative surveillance regardless of approach. Regular monitoring of renal function and aortic morphology is essential for longevity of the repair. Following endovascular repair, additional care and concern for endograft integrity and the development of endoleaks remains paramount.^8^

**Fig 3:**
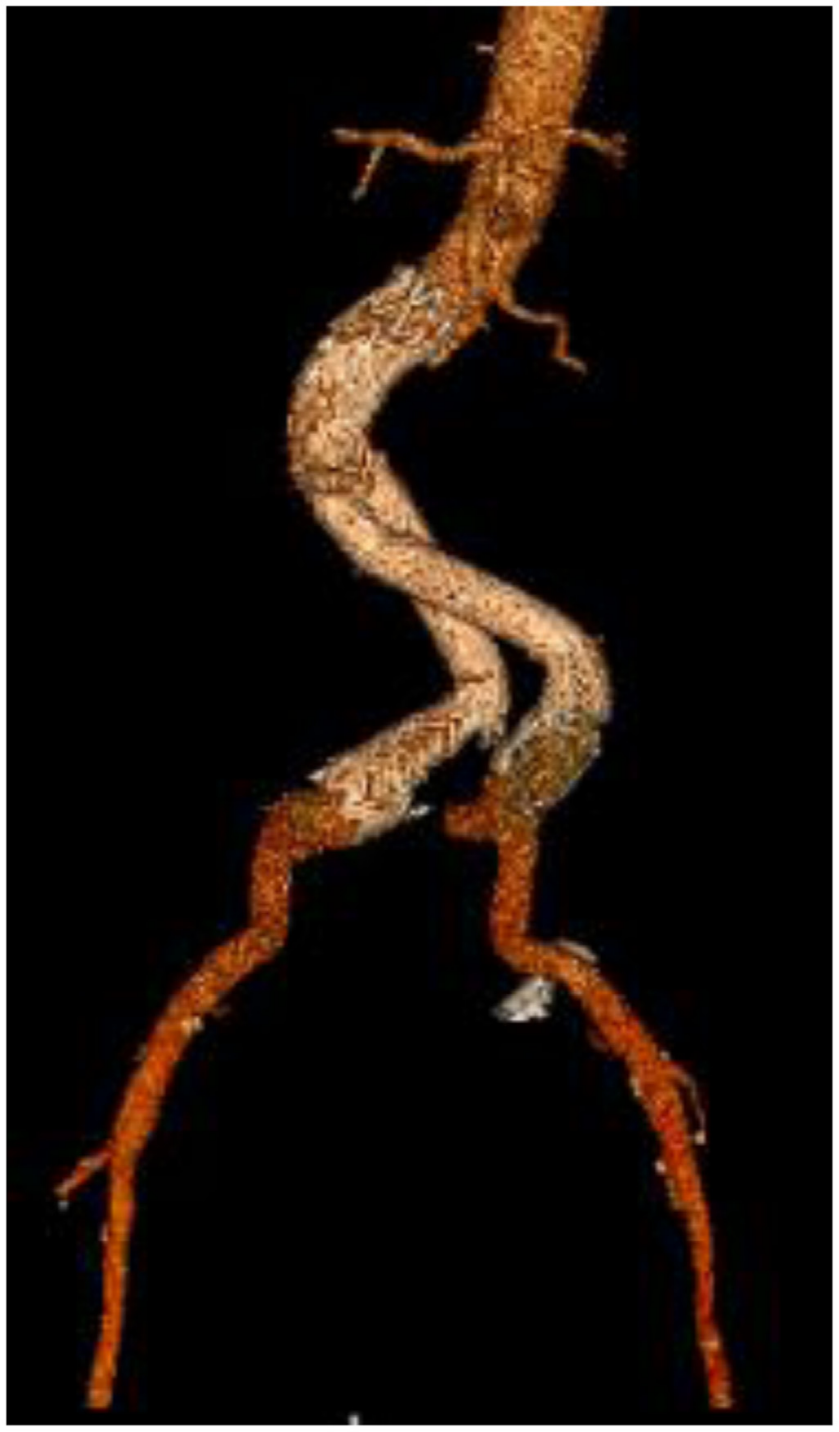
Computed tomography (*CT*) reconstruction of endograft at 1-year surveillance appointment.

**Fig 4:**
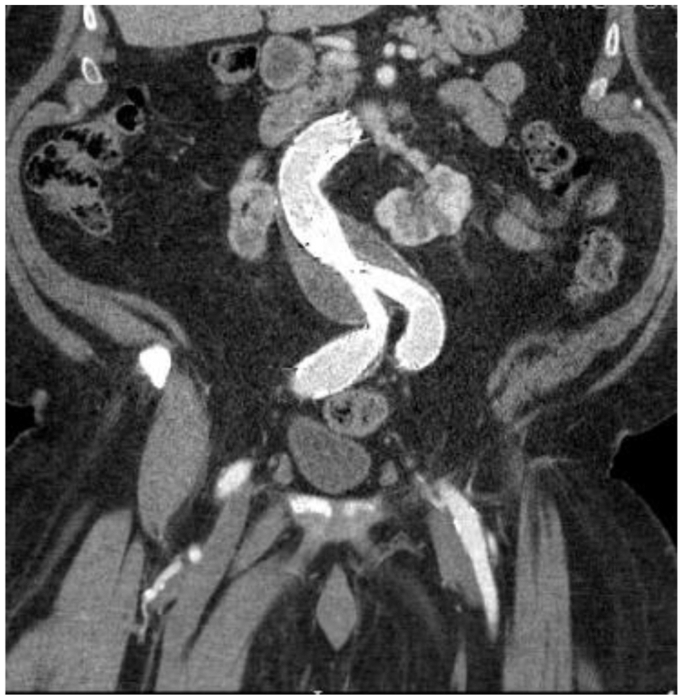
Coronal computed tomography (*CT*) imaging of patent endograft at 1-year surveillance appointment.

In the case presented, preoperative planning including CT angiography, duplex, conventional angiography, and split renal function testing, which demonstrated type II vasculature (Fig 2) with a left and right renal artery and a dominant short anterior artery arising from the aorta (Fig 1, *A–F*). Given the underlying comorbidities, aortic morphology, and renal artery anatomy, the safest option was felt to be an endovascular repair with visceral snorkel (Fig 1, *G–H*). The patient has been surveilled at 1, 3, and 6 months. The latest follow-up was at 12 months, with both CT and arterial duplex studies showing widely patent grafts without evidence of endoleak and preserved renal function (Figs 3 and 4).

## Conclusion

AAA in the setting of HSK is a rare and complex aortic pathology. Methodical planning is essential to ensure aneurysm exclusion, preservation of renal function, and minimizing morbidity and mortality. In the elective setting, open, hybrid, and endovascular repairs are all feasible options with their own advantages and disadvantages. Due to the patient’s comorbidities and morbid obesity, we elected to employ an endovascular approach with central renal snorkel, demonstrating the versatility of endovascular repair in the setting of complex aortic pathologies to minimize operative risk.

## Author Contributions

Conception and design: EM, SF, NR, TB

Analysis and interpretation: EM, SF, NR, TB

Data collection: EM

Writing the article: EM, SF

Critical revision of the article: EM, SF, NR, TB

Final approval of the article: EM, SF, NR, TB

Statistical analysis: Not applicable

Obtained funding: Not applicable

Overall responsibility: EM

## Disclosures

None.
